# Long-term Safety in Epstein–Barr Virus–Seropositive Kidney-only Transplant Recipients Treated With Belatacept in Clinical Practice: Final Study Results From the ENLiST Registry

**DOI:** 10.1097/TXD.0000000000001644

**Published:** 2024-05-17

**Authors:** Christian P. Larsen, Flavio Vincenti, Tzuyung D. Kou, Craig A. Shadur, Barbara Bresnahan, Stanley C. Jordan, E. Steve Woodle, Nelson Goes, John Vella, David Wojciechowski, Martin S. Polinsky, Andres Gomez-Caminero

**Affiliations:** 1 Department of Surgery, Emory University Transplant Center, Atlanta, GA.; 2 Departments of Medicine and Surgery, University of California, San Francisco, Transplant Center, San Francisco, CA.; 3 Worldwide Patient Safety, Bristol Myers Squibb, Princeton, NJ.; 4 Transplantation Service, Iowa Kidney Physicians, Des Moines, IA.; 5 Division of Nephrology, Department of Medicine, Medical College of Wisconsin, Milwaukee, WI.; 6 Comprehensive Transplant Center, Cedars Sinai, Los Angeles, CA.; 7 Department of Surgery, University of Cincinnati, Cincinnati, OH.; 8 Kidney Transplant Clinics, Kaiser Permanente, San Francisco, CA.; 9 Division of Nephrology and Transplantation, Maine Nephrology Associates, Portland, ME.; 10 Department of Internal Medicine, The University of Texas Southwestern Medical Center, Dallas, TX.; 11 Research and Development/Global Drug Development, Bristol Myers Squibb, Princeton, NJ.; 12 Worldwide Health Economic and Outcomes Research, Bristol Myers Squibb, Princeton, NJ.

## Abstract

**Background.:**

Belatacept, a selective T-cell costimulation blocker, was associated with improved survival and renal function but also with a risk of posttransplant lymphoproliferative disorder (PTLD) in adult kidney transplant recipients in phase 3 trials. This registry examined long-term safety in Epstein–Barr virus (EBV)–seropositive kidney transplant recipients treated with belatacept.

**Methods.:**

This US-based, prospective, voluntary, multicenter registry (Evaluating Nulojix Long-Term Safety in Transplant [ENLiST]) included adult EBV-seropositive kidney-only transplant recipients treated de novo (within 14 d of transplantation) with belatacept. Primary objectives were to estimate incidence rates of confirmed PTLD, central nervous system (CNS) PTLD, and progressive multifocal encephalopathy (PML). The minimum follow-up was 2 y.

**Results.:**

Of 985 enrolled transplant recipients, 933 EBV-seropositive patients received belatacept, with 523 (56.1%) receiving concomitant tacrolimus at transplant (for up to 12 mo). By study end, 3 cases of non-CNS PTLD (incidence rate, 0.08/100 person-years), 1 case of CNS PTLD (0.03/100 person-years), and no cases of PML had been reported. Two patients with non-CNS PTLD received concomitant belatacept and tacrolimus and 1 received belatacept and lymphocyte-depleting therapy. Incidence rates were comparable between patients who received concomitant belatacept and tacrolimus and those who did not receive tacrolimus (0.09/100 person-years and 0.07/100 person-years, respectively; *P* = 0.96). Two of 4 patients with PTLD died, and 2 were alive at the end of the study. Cumulatively, 131 graft losses or deaths were reported by study end.

**Conclusions.:**

Our results from the ENLiST registry, a large, prospective real-world study, showed that the incidence rates of PTLD and CNS PTLD in belatacept-treated EBV-seropositive transplant recipients were consistent with findings from previous phase 3 trials.

Calcineurin inhibitor (CNI)-based immunosuppressive regimens have resulted in low rates of early rejection and have long been the standard of care for immunosuppression in kidney transplant recipients.^1^ However, CNIs are potentially nephrotoxic and may contribute to the progressive loss of allograft function over time, thereby reducing long-term survival.^2^ Currently, >85% of kidney transplant recipients in the United States receive a tacrolimus-based regimen following transplantation.^3^ In an analysis of data from the US Scientific Registry of Transplant Recipients collected between 1995 and 2017, the 1-y renal allograft survival rate was reported to be 97.8% for transplants from living donors and 94.3% for transplants from deceased donors, with rates decreasing to 88.0% and 78.1%, respectively, at 5 y posttransplantation.^4^

Belatacept, a fusion protein composed of a modified Fc fragment from a human immunoglobulin G1 molecule linked to the modified extracellular domain of cytotoxic T-lymphocyte–associated antigen 4, selectively inhibits T-cell activation through blockade of the CD28–CD80/86 costimulatory pathway.^5^ Belatacept was approved in 2011 in the United States and European Union for the prophylaxis of organ rejection in kidney transplant recipients^6,7^ based on data from 2 phase 3 clinical trials: BENEFIT (Belatacept Evaluation of Nephroprotection and Efficacy as First-Line Immunosuppression Trial) and BENEFIT-EXT (Extended Criteria Donors).^8,9^ In both trials, belatacept treatment of kidney-only transplant recipients was associated with similar patient and graft survival, an improved cardiovascular and metabolic profile, a reduced incidence of chronic allograft nephropathy, and superior kidney function compared with cyclosporine treatment.^8–11^ In patients who received living or standard criteria deceased donor transplants, belatacept treatment also was associated with a long-term (through 7 y posttransplant) reduced risk of graft loss or death compared with cyclosporine treatment.^10^

Posttransplant lymphoproliferative disorder (PTLD) is an infrequent but severe complication of transplantation that develops as a result of uncontrolled B-cell proliferation because of blunted immunological surveillance.^12^ In an analysis of data from the US Organ Procurement and Transplantation Network database collected from 1999 to 2008, the PTLD incidence rate among kidney transplant recipients was reported to be 1.58 per 1000 person-years.^13^ PTLD is associated with Epstein–Barr virus (EBV) infection; 60% to 80% of all patients with PTLD are also seropositive for EBV.^14^ The lack of immunity to EBV at the time of transplantation, concomitant cytomegalovirus (CMV) infection, and the use of lymphocyte-depleting therapies are known risk factors for the development of PTLD in kidney transplant recipients.^15,16^ In BENEFIT and BENEFIT-EXT, the approved dosing regimen of belatacept was associated with a PTLD incidence rate of 0.2 to 0.25 per 100 person-years in EBV-seropositive patients^10,11^ and 0 to 5.19 per 100 person-years in EBV-seronegative patients.^11^ There were 2 cases of PTLD with central nervous system (CNS) involvement in BENEFIT^8^ and 5 cases of CNS PTLD in BENEFIT-EXT; however, 5 of these 7 cases occurred with the more intensive belatacept dosing regimen (involving higher dosing during the first 6 mo posttransplant relative to the approved regimen) and in patients who were EBV-seronegative.^11,17^ For comparison, the incidence rate of PTLD in the cyclosporine group was 0.1 to 0.14 per 100 person-years in EBV-seropositive patients and 0 to 0.6 per 100 person-years in EBV-seronegative patients, with no case of CNS PTLD reported.^10,11^ Because of the increased risk of PTLD in EBV-seronegative patients, belatacept is indicated for use only in EBV-seropositive kidney transplant recipients.^6,7^

Progressive multifocal leukoencephalopathy (PML) is a rare and serious brain infection caused by the John Cunningham virus. PML was reported in 1 renal transplant patient in BENEFIT-EXT and 1 liver transplant patient in a phase 2 trial.^18^ Both of these patients had received belatacept at the more intensive dosing regimen.^6,9,17^

In the postapproval setting, real-world studies have been increasingly used to guide clinical decision-making, complement results from randomized clinical trials, assess treatment patterns, and evaluate safety in a larger patient population.^19^ Given the complexities and individualized nature of the immunosuppressive regimens used in the real-world setting, the risk of PTLD and PML in belatacept-treated patients requires further evaluation. To this end, we used a patient registry to assess the incidence rates of PTLD, including PTLD with CNS involvement, and PML in adult EBV-seropositive kidney transplant recipients treated with belatacept in routine clinical practice.

## MATERIALS AND METHODS

### Patient Population

**FIGURE 1.**
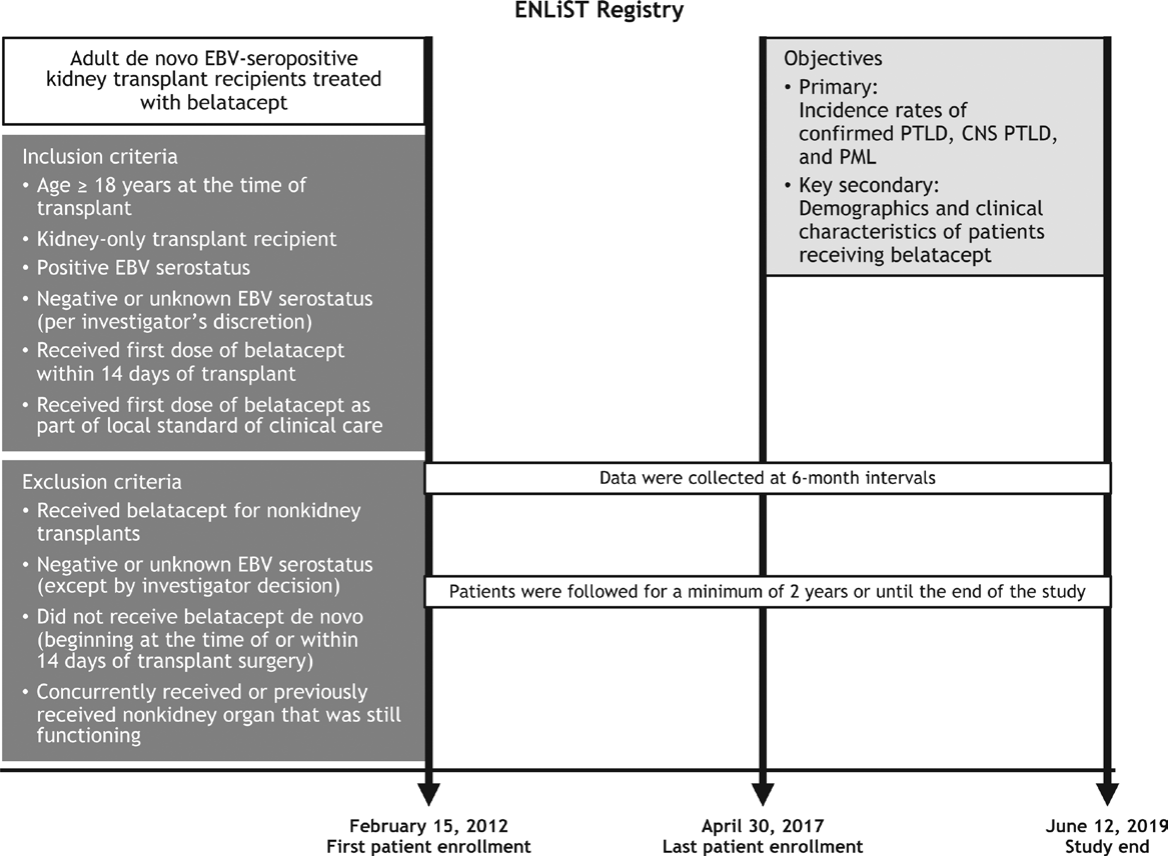
Study design. CNS, central nervous system; EBV, Epstein–Barr virus; PML, progressive multifocal leukoencephalopathy; PTLD, posttransplant lymphoproliferative disorder.

This prospective, voluntary, multicenter registry study (Evaluating Nulojix Long-Term Safety in Transplant [ENLiST]; NCT01386359) included adult EBV-seropositive kidney-only transplant recipients treated de novo with belatacept (defined as administration of belatacept beginning within 14 d of renal transplantation) in routine clinical practice in the United States. At the start of the study, patients who were EBV-seropositive were the primary focus, but 9 patients who were EBV-seronegative or whose serostatus was unknown also were enrolled. One year after the start of the study, the protocol was amended based on the US Food and Drug Administration guidance to exclude EBV-seronegative patients. Although efforts were made to enroll de novo patients during their transplant hospitalization, per protocol, transplant patients treated with belatacept before implementation of the ENLiST registry could also be retrospectively enrolled into the registry. Patients were followed for a minimum of 2 y or until the end of the study (Figure 1). Written informed consent was obtained at the time of enrollment. This study was conducted in accordance with Good Clinical Practice per the International Conference on Harmonisation and applicable regulatory requirements. The study protocol, including amendments, was reviewed and approved by the Institutional Review Board/Independent Ethics Committee for each site before study initiation.

### Data Collection

Data were entered by participating centers into a computerized data system using structured electronic case report forms. Patient data were collected at enrollment and at 6-mo intervals from the time of the first belatacept dose until the end of the study. The occurrence of PTLD, CNS PTLD, and PML was confirmed from medical records. Clinical information; laboratory, imaging, and pathology results; and tissue biopsy slides for reported cases of PTLD and PML were reviewed by an independent panel consisting of ≥3 experts to confirm and further document the cases. Cases of primary CNS PTLD were confirmed by histopathologic or cytologic examination of tissue from CNS lesions that documented the presence of a cerebral lymphoma in the absence of radiological evidence of lesions suspicious for PTLD outside of the CNS.

### Study Objectives

The primary objectives of this study were to determine the incidence rates of confirmed non-CNS PTLD, CNS PTLD, and PML in adult EBV-seropositive kidney transplant recipients treated de novo with belatacept. Secondary objectives included describing the demographic and clinical characteristics of the study population, estimating patient and graft survival rates, and determining the incidence rates of confirmed non-CNS PTLD, CNS PTLD, and PML by baseline CMV serostatus. Examining the incidence rates of confirmed non-CNS PTLD, CNS PTLD, and PML in the context of concomitant immunosuppressive therapies and comparing the incidence rates of PTLD and PML in the ENLiST registry with those reported in previous belatacept clinical trials were exploratory objectives.

### Statistical Analysis

Incidence of non-CNS PTLD, CNS PTLD, and PML, and patient and graft survival were estimated by dividing the number of cases by the accumulated person-years at risk. The person-time at risk for each patient was estimated from the date of the first belatacept dose up to the date of the event or last follow-up, whichever occurred first, and was confined to treatment within 28 d of the last belatacept dose. The 95% confidence intervals (CIs) were estimated assuming a Poisson distribution of the events. Incidence rates were stratified by other therapies used during immunosuppression. Descriptive statistics were used to summarize the clinical and demographic characteristics of study participants.

## RESULTS

### Patients

**FIGURE 2.**
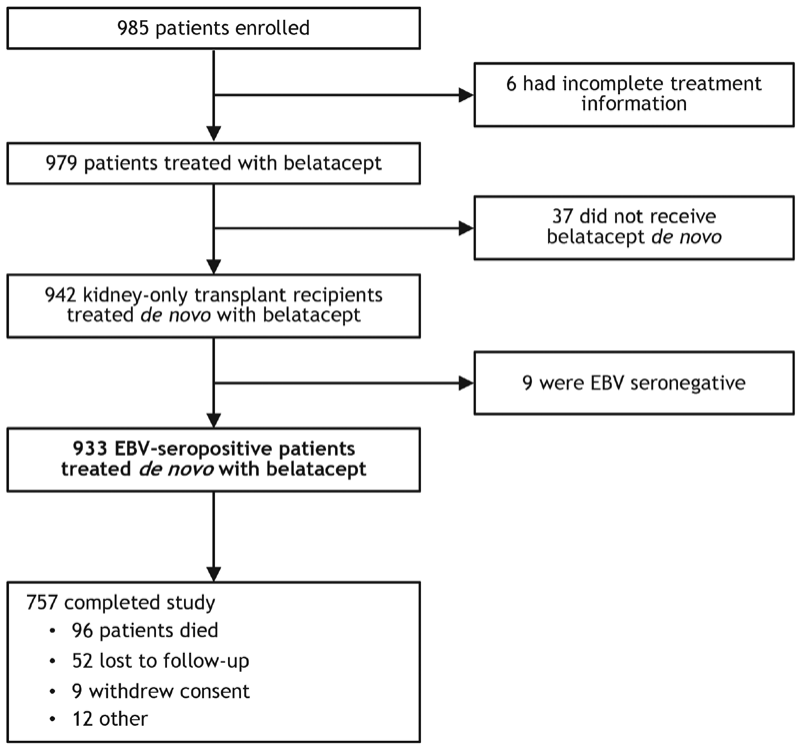
Patient disposition. EBV, Epstein–Barr virus.

Between February 15, 2012 (date of first patient enrollment) and April 30, 2017 (date of last patient enrollment), 985 kidney transplant recipients were enrolled at 35 transplant centers in the United States. Among the enrolled patients, 933 EBV-seropositive patients were treated de novo with belatacept (beginning within 14 d of transplantation) and analyzed (Figure 2). As to the 52 excluded patients, 6 had incomplete treatment information, 37 did not receive belatacept de novo, and 9 were EBV-seronegative. The study end date was June 12, 2019, at which time all 933 patients were discontinued from follow-up. Median follow-up duration was 1581 d (interquartile range, 1066–2131).

The baseline characteristics of the study participants are shown in Table 1; the mean age was 52.6 y, 63.2% were male, 54.0% were White, 64.8% were CMV-seropositive, and 95.3% were receiving CMV prophylaxis. At transplant, 56.1% of patients received concomitant belatacept and tacrolimus, with tacrolimus then tapered to discontinuation over the next 10–12 mo; 25.4% of patients received concomitant belatacept and lymphocyte-depleting therapy.

**TABLE 1.** Baseline demographics of EBV-seropositive kidney-only transplant recipients treated de novo with belatacept

Characteristic	Belatacept (N = 933)
Age at transplant, mean (SD), y	52.6 (13.4)
Male, n (%)	590 (63.2)
Race, n (%)	
White	504 (54.0)
Black	349 (37.4)
Asia	33 (3.5)
Other	47 (5.0)
Body mass index, n (%)	
<20	41 (4.4)
20–25	236 (25.3)
26–30	317 (34.0)
>30	335 (35.9)
Not reported	4 (0.4)
Recipient CMV antibody serostatus, n (%)	
Positive	605 (64.8)
Negative	324 (34.7)
Unknown	3 (0.3)
Not reported	1 (0.1)
Recipient JCV antibody serostatus, n (%)	
Positive	2 (0.2)
Negative	2 (0.2)
Unknown	928 (99.5)
Not reported	1 (0.1)
CMV prophylaxis, n (%)	
Yes	889 (95.3)
No	44 (4.7)
Initial immunosuppressive therapy, n (%)	
Belatacept	885 (94.9)
Belatacept plus tacrolimus	523 (56.1)
Belatacept plus lymphocyte-depleting therapy^*a*^	237 (25.4)

^*a*^Alemtuzumab, antithymocyte globulin (ATGAM or Thymoglobulin), or OKT3.

CMV, cytomegalovirus; JCV, John Cunningham virus.

The baseline characteristics of the donor kidneys are shown in Table 2; 37.9% were from living donors, and 40.7% were from standard criteria deceased donors. The mean donor age was 41 y, 44.5% were male, 84.0% were EBV-seropositive, and 59.6% were CMV-seropositive.

**TABLE 2.** Baseline demographics of kidney donors

Characteristic	Belatacept (N = 933)
Age at transplant donation, mean (SD), y	41.0 (15.3)
Sex, n (%)	
Male	415 (44.5)
Female	454 (48.7)
Not reported	64 (6.9)
Donor EBV antibody serostatus, n (%)	
Positive	784 (84.0)
Negative	60 (6.4)
Unknown	89 (9.5)
Donor CMV antibody serostatus, n (%)	
Positive	556 (59.6)
Negative	350 (37.5)
Unknown	27 (2.9)
Transplant donor type, n (%)	
Living, related	163 (17.5)
Living, unrelated	191 (20.5)
Deceased	576 (61.7)
Standard criteria donor^*a*^	380 (66.0)
Extended criteria donor^*a*^	80 (13.9)
Donation after cardiac death^*a*^	70 (12.2)
Not reported^*a*^	46 (8.0)
Not reported	3 (0.3)

^*a*^Percentages are based on the number of deceased donors.

CMV, cytomegalovirus; EBV, Epstein–Barr virus.

The mean duration of belatacept exposure was 43.2 mo (Table 3). The majority of patients (84.0%) received belatacept for at least 12 mo.

**TABLE 3.** Extent of belatacept exposure

	Belatacept (N = 933)
Belatacept exposure,^*a*^ n (%), mo	
0–3	48 (5.1)
3–6	43 (4.6)
6–9	37 (4.0)
9–12	21 (2.3)
12–24	71 (7.6)
24–36	147 (15.8)
36–48	151 (16.2)
>48	415 (44.5)
Duration of treatment, mean (SD), mo	43.2 (25.2)

^*a*^Defined as the last infusion date − the first infusion date + 28 d.

### Safety

**FIGURE 3.**
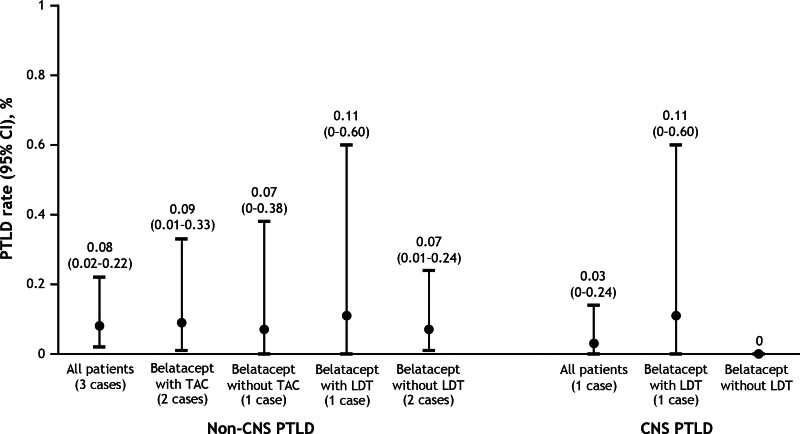
Incidence rates of CNS and non-CNS PTLD in EBV-seropositive kidney-only transplant recipients treated de novo with belatacept. CNS, central nervous system; EBV, Epstein–Barr virus; LDT, lymphocyte-depleting therapy; PTLD, posttransplant lymphoproliferative disorder; TAC, tacrolimus.

By the end of the study, 3 cases of non-CNS PTLD had been reported, with 2 occurring in the first year and 1 occurring in the second year. The incidence rates for non-CNS PTLD were 0.22 (95% CI, 0.03-0.79) per 100 person-years in the first year and 0.11 (95% CI, 0-0.64) per 100 person-years in the second year. The cumulative incidence rate for non-CNS PTLD was 0.08 (95% CI, 0.02-0.22) per 100 person-years (Figure 3). One case of CNS PTLD was reported >1 y posttransplant; the incidence rate of CNS PTLD during the second year was 0.11 (95% CI, 0-0.64) per 100 person-years. The cumulative incidence rate for CNS PTLD was 0.03 per 100 person-years (Figure 3). No case of PML was reported.

Two patients with non-CNS PTLD received concomitant belatacept and tacrolimus at transplant, and 1 patient with non-CNS PTLD received concomitant belatacept and lymphocyte-depleting therapy (Table 4). Incidence rates for non-CNS PTLD were similar for patients who received concomitant belatacept and tacrolimus and those who received belatacept without tacrolimus at transplant (0.09 and 0.07 per 100 person-years, respectively; *P* = 0.96). Similarly, no difference in incidence rates was observed between patients treated with concomitant belatacept and lymphocyte-depleting therapy and those treated with belatacept without lymphocyte-depleting therapy (0.11 and 0.07 per 100 person-years, respectively). The single patient who developed CNS PTLD had received concomitant belatacept and lymphocyte-depleting therapy.

**TABLE 4.** Incidence rates of non-CNS and CNS PTLD in EBV-seropositive kidney-only transplant recipients treated de novo with belatacept

	Patients, n	Patients with PTLD, n	Overall event rate (95% CI), %	Incidence rate per 100 person-years (95% CI)
Non-CNS PTLD				
All patients	933	3	0.32 (0.10-0.90)	0.08 (0.02-0.22)
Belatacept with tacrolimus	523	2	0.38 (0.00-1.40)	0.09 (0.01-0.33)
Belatacept without tacrolimus	410	1	0.24 (0.01-1.36)	0.07 (0.00-0.38)
Belatacept with LDT	237	1	0.40 (0.00-2.30)	0.11 (0.00-0.60)
Belatacept without LDT	696	2	0.30 (0.00-1.00)	0.07 (0.01-0.24)
CNS PTLD				
All patients	933	1	0.10 (0.00-0.60)	0.03 (0.00-0.14)
Belatacept with LDT	237	1	0.40 (0.00-2.30)	0.11 (0.00-0.60)
Belatacept without LDT	696	0	0	0

CI, confidence interval; CNS, central nervous system; EBV, Epstein–Barr virus; LDT, lymphocyte-depleting therapy; PTLD, posttransplant lymphoproliferative disorder.

Because of the lower than anticipated PTLD event rates, incidence rates by baseline CMV serostatus were not calculated. Multivariate regression analysis failed to identify any factors significantly related to PTLD risk, a possible consequence of the low number of observed PTLD events (data not shown). Among the 37 patients who did not receive belatacept de novo, no case of PTLD or PML was reported. In addition, no case of PTLD or PML was reported among the 9 EBV-seronegative patients. Of the 4 patients with PTLD, 2 had died during the study and 2 were alive at the end of the study.

### Graft Loss or Death

**FIGURE 4.**
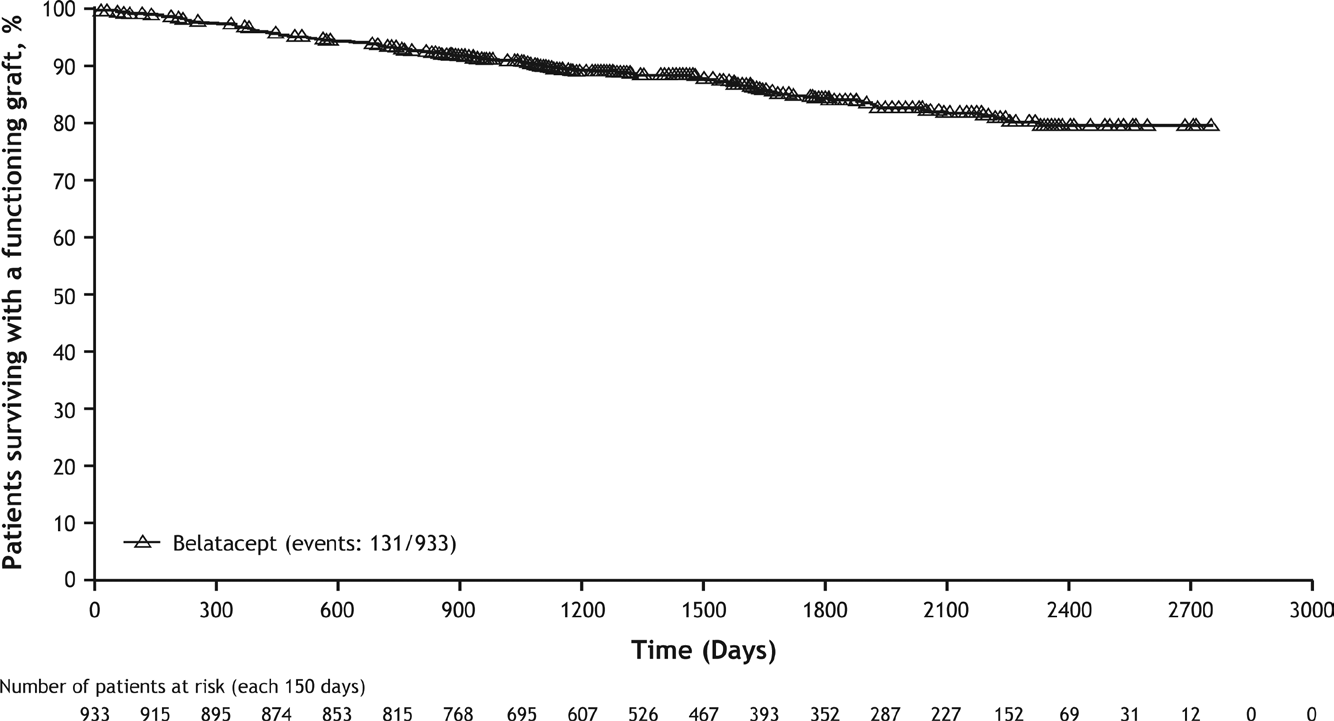
Kaplan–Meier estimated time to graft loss or death in EBV-seropositive kidney-only transplant recipients treated de novo with belatacept. EBV, Epstein–Barr virus.

By the end of the study, 131 deaths or graft losses had been reported among 933 EBV-seropositive patients, corresponding to a Kaplan–Meier estimated graft survival rate of 80% at 7 y (Figure 4). By the end of the study, a total of 57 graft losses and 96 deaths were reported; 74 patients died with functioning grafts.

## DISCUSSION

The ENLiST registry is the largest prospective real-world study to date to evaluate the occurrence of PTLD and PML in kidney transplant recipients treated de novo with belatacept. The cumulative incidence rates of non-CNS PTLD (0.08 per 100 person-years) and CNS PTLD (0.03 per 100 person-years) among EBV-seropositive patients in this registry were consistent with those reported in prior belatacept trials.^10,11,17^ Among EBV-seropositive patients in BENEFIT and BENEFIT-EXT, the overall incidence rate of PTLD was 0.2–0.25 per 100 person-years in patients receiving the approved belatacept dosing regimen and 0.1–0.14 per 100 person-years in patients receiving cyclosporine.^10,11^ In a meta-analysis involving 1535 renal transplant recipients from 5 randomized studies, the relative risk of PTLD was found to be similar for belatacept- and CNI-treated patients (risk ratio, 2.79; 95% CI, 0.61-12.66).^20^ In a pooled safety analysis involving renal transplant recipients participating in 3 clinical studies of belatacept, PTLD was reported in 1.5% (14/953) of belatacept-treated patients and 0.4% (2/476) of cyclosporine-treated patients.^17^ Nine of the 14 cases of PTLD observed in belatacept-treated patients involved the CNS; further analyses revealed that the risk of CNS PTLD was lower in patients who were EBV-seropositive (0.5% [4/805]) versus seronegative (5.2% [5/96]) and in those receiving the approved (0.6% [3/472]) versus the more intensive (1.3% [6/477]) belatacept dosing regimen.

No case of PML was reported in the ENLiST registry, which only included patients administered the approved belatacept dosing regimen. This serious infection is an important safety concern. Of the 2 cases of PML reported in BENEFT-EXT, 1 occurred in a patient who received the more intensive belatacept dosing regimen and the other occurred in a patient treated with cyclosporine.^7,9^ Although mycophenolate mofetil has been associated with PML,^21^ the risk of this viral infection with biologics and concomitant immunosuppressive regimens remains poorly understood because of its complex pathogenesis.^22^ However, because PML is invariably fatal, it is important to continually monitor patients for disease signs/symptoms and to consider risk-mitigating strategies.

Approximately 25% (237/933) of patients in ENLiST received lymphocyte-depleting therapy, a known risk factor for PTLD,^16^ yet the overall number of PTLD cases in this subset of patients was low (non-CNS PTLD, n = 1; CNS PTLD, n = 1). Similarly, use of concomitant tacrolimus had no effect on the rate of PTLD, which occurred in 2 of 523 tacrolimus-treated patients. Notably, in both BENEFIT and BENEFIT-EXT, where basiliximab (a nonlymphocyte-depleting monoclonal antibody that blocks T-cell activation)^23^ was used as induction therapy, the incidence rates of PTLD were comparable to that reported in ENLiST.^10,11^

Baseline demographics and clinical characteristics of transplant recipients in this registry were generally similar to those reported in BENEFIT and BENEFIT-EXT, although some differences were observed.^8,9^ Registry participants were older than patients enrolled to BENEFIT (mean of 52.6 versus 43.2 y) but were closer in age to those enrolled to BENEFIT-EXT (mean of 56.2 y). In ENLiST, 13.9% of patients received transplants from extended criteria donors, whereas all BENEFIT study participants were recipients of living or standard criteria deceased donor kidneys,^8^ and all BENEFIT-EXT study participants received extended criteria donor kidneys, specifically those from deceased donors who met United Network for Organ Sharing extended criteria for donation,^24^ those from non–heart-beating donors, or those with prolonged (>24 h) estimated cold ischemia times.^9^ Irrespective of donor type and recipient baseline characteristics, PTLD incidence rates in belatacept-treated kidney transplant recipients were similar across ENLiST, BENEFIT, and BENEFIT-EXT.

In ENLiST, the Kaplan–Meier estimated rate of survival with a functioning graft at 7 y was 80%. Because this was a voluntary registry, this should be considered an approximation only because follow-up data could not be obtained for several patients. However, this estimated graft/survival rate is within the range observed at 7 y posttransplant for belatacept-treated participants in BENEFIT (87.2%) and BENEFIT-EXT (65.3%).^10,11^

Our study had several limitations. First, information regarding patient comorbidities and donor–recipient human leukocyte antigen matching was incomplete. Second, minimal data were available regarding immunosuppressive medications (other than belatacept). Third, and as mentioned earlier, several patients were lost to follow up (n = 52), preventing an accurate assessment of cumulative graft/patient survival. Finally, although the study did not incorporate a central pathology review, all cases of PTLD (and their outcomes) were adjudicated by an independent expert panel to ensure their accuracy.

In conclusion, this real-world study showed that the risk of PTLD and CNS PTLD, as well as PML, remains low in EBV-seropositive kidney transplant recipients treated de novo with belatacept in routine clinical practice. This study is unique in the size of the EBV-seropositive patient population (n = 933) and its prospective design for collection of real-world evidence. Our observations are consistent with the benefit–risk profile of belatacept reported in the phase 3 BENEFIT and BENEFIT-EXT trials.

## ACKNOWLEDGMENTS

The authors would like to acknowledge Kathleen Soucek, MS, for her contribution as the protocol manager and Mary L Skovron, DrPH, for her contribution as the director of this study. Medical writing and editorial assistance were provided by Kakoli Parai, PhD, Vasupradha Vethantham, PhD, and Michele Salernitano, of Ashfield MedComms, an Inizio company, and were funded by Bristol Myers Squibb.
